# Staphylococcal skin infection isolates from dogs without recent antibiotic exposure are 100% susceptible to clindamycin

**DOI:** 10.3389/fvets.2024.1512582

**Published:** 2025-02-10

**Authors:** W. Cooper Brookshire, Larry D. Ballard, Vernon C. Langston, Joo Youn Park, Keun-Seok Seo

**Affiliations:** ^1^Department of Clinical Sciences, College of Veterinary Medicine, Mississippi State University, Mississippi State, MS, United States; ^2^Department of Pathobiology and Population Medicine, College of Veterinary Medicine, Mississippi State University, Mississippi State, MS, United States; ^3^Department of Comparative Biomedical Sciences, College of Veterinary Medicine, Mississippi State University, Mississippi State, MS, United States

**Keywords:** veterinary, antibiogram, *Staphylococcus pseudintermedius*, *S. schleiferi* antibiotic resistance, bacterial folliculitis, pyoderma, canine

## Abstract

The objective of this study was to create an antibiogram representative of bacterial skin infections in canine patients that would typically be treated empirically, i.e., without risk factors for antibiotic resistance, such as a history of recent antibiotic use, antibiotic treatment failure, or recurrent infections. Traditional antibiograms are a form of passive surveillance and report antibiotic susceptibility of isolates from a specific laboratory, hospital, or region for a given period of time. However, traditional antibiograms are biased towards more resistance, because infections that have antibiotic susceptibility tests are more likely to be resistant, due to risk factors such as recent antibiotic treatment, hospitalization, or a history of previous antibiotic-resistant infections. Antibiotic susceptibility testing was performed on 67 pathogenic canine staphylococcal isolates (62 *Staphylococcus pseudintermedius* and 5 *Staphylococcus schleiferi*) from patients who met the study inclusion criteria, and 100% of isolates were susceptible to antibiotics commonly prescribed for canine staphylococcal skin infections, including clindamycin. Additionally, a subset of 49 isolates were also susceptible to chlorhexidine. The isolates were susceptible to a very low concentration of chlorhexidine, which supports its use as a preferred topical treatment. These data strongly indicate that dogs without a history of recent antibiotic use, treatment failure, or recurrent infections that present with bacterial skin infections are at low risk of antibiotic resistance. If systemic antibiotics are indicated in these patients with this clinical history and presentation, clindamycin should be considered as first-line therapy, owing to its 100% susceptibility in this antibiogram and less selection pressure for antibiotic resistant bacteria, compared to alternatives such as cephalosporins.

## 1 Introduction

Infections caused by antimicrobial resistant bacteria are emerging as serious threats in veterinary medicine due to the emergence of carbapenemase and extended-spectrum *β*-lactamase (ESBL) producing Enterobacterales and methicillin-resistant Staphylococci. In human medicine, it is estimated that up to 50% of antibiotic prescriptions in U.S. are either unnecessary or inappropriate (1). Accurate statistics are unavailable for companion animal medicine, but it is likely that unnecessary or inappropriate prescriptions are dispensed at a similar rate (2). A survey of 654 veterinarians indicated that 94% of respondents choose antibiotic therapy empirically when presented with acute skin infections (pyoderma) (3). Empirical “best guess” therapy, unfortunately, carries a significant risk of inappropriate antibiotic selection. Compiled databases of previous culture results, called antibiograms, are commonly utilized to improve empiric antibiotic prescribing decisions. However, these databases are inherently flawed, because they often represent the type of recurrent, refractory, or otherwise severe infections that are more likely to be resistant, rather than the common types of more straightforward infections that veterinarians are treating empirically in practice (4).

The International Society for Companion Animal Infectious Diseases (ISCAID) recently emphasized that there is a need for improved antimicrobial stewardship both in veterinary hospitals and practices. Recently published longitudinal data suggests that many veterinarians adopt stewardship recommendations (5). Many authors have published data describing how veterinarians make prescribing decisions (6, 7). There is, however, a very distinct void in the veterinary literature describing antibiotic sensitivity patterns specifically for patients with infections that would normally be treated empirically. Ironically, the most common infections in veterinary medicine are usually treated empirically (5, 8–10). A limited number of studies have described antibiotic susceptibility of patients that would typically be treated empirically. Larsen et al. performed a study in Denmark to compare the antibiotic susceptibility patterns of antibiotic naive patients with skin infections to patients with an unknown clinical history; bacteria isolated from antibiotic naive patients were less likely to be resistant compared to patients with an unknown clinical history (4). Similarly, Clare et al. recently published a study comparing treatment regimens for uncomplicated urinary tract infections in patients with no history of antibiotic use in the prior 3 months. Interestingly, 100% of isolates were susceptible to potentiated sulfonamides and 1st generation cephalosporins (11). These data and the association between no recent antibiotic therapy and antibiotic susceptibility strongly support the development of antibiograms representative of infections typically treated empirically. Based on these data, we suspect that infections typically treated empirically have resistance patterns that will have a meaningful effect on antibiotic stewardship recommendations.

Superficial bacterial folliculitis (herein, pyoderma) has been identified as the most common type of canine skin infection, which is the principal use for antimicrobials in small animal practice (4). The predominant pathogenic bacterial species associated with pyoderma is *Staphylococcus pseudintermedius* (*S. pseudintermedius*)*. Staphylococcus schleiferi* (*S. schleiferi*) is also suspected to be pathogenic and associated with pyoderma. Other bacterial species, such as coagulase negative staphylococcal species (e.g., *Staphylococcus epidermidis*), *Streptococcus canis*, and *Pseudomonas aeruginosa* are occasionally isolated on bacterial cultures, but the role these organisms play in pathogenicity is unclear (12). *Staphylococcus pseudintermedius* and *S. schleiferi* will, herein, be referred to as “pathogenic staphylococcal species/isolates,” and be the primary focus of this article. The Antimicrobial Guidelines Working Group of ISCAID states that bacterial culture and sensitivity for bacterial pyoderma is “never contraindicated” (12). However, empiric therapy for superficial bacterial pyoderma remains very common. A study published in 2014 suggests that only 2.4% of primary care vets in the United Kingdom perform culture and sensitivity when treating superficial bacterial pyoderma (8).

There are many systemic antibiotics that are commonly used to treat canine pyoderma. Surveys from Australia, New Zealand, Canada, and the United Kingdom suggest that cephalosporins (cephalexin, cefpodoxime) and amoxicillin/clavulanic acid are the most commonly utilized antibiotics (3, 5, 8–10). However, clindamycin is considered by many authors to be a preferred first line drug because it is well tolerated by dogs, effective against infections with susceptible bacteria, and has a more narrow spectrum of antibacterial activity. Clindamycin has little to no effect on many Enterobacterales, unlike cephalexin and cefpodoxime, which strongly select for resistance in Enterobacterales. Additionally, clindamycin is less likely to select for methicillin resistant staphylococcus, when compared to beta lactam antibiotics such as cephalexin and cefpodoxime (4, 13, 14).

Chlorhexidine gluconate is well documented as a highly effective topical treatment for canine pyoderma (15). The ISCAID working group states that chlorhexidine topical therapy for superficial bacterial pyoderma is probably underutilized despite its advantages that include rapid resolution of clinical signs and reduced duration of antimicrobial therapy (4). Evaluation of *in-vitro* antimicrobial susceptibility of topical used therapies is complex, since traditional breakpoints are unavailable. The generally accepted chlorhexidine epidemiologic minimal inhibitory concentration (MIC) cutoff value for *Staphylococcus aureus* is 8 μg/mL, which is the cutoff value we chose to use for *S. pseudintermedius* and *S. schleiferi* to distinguish between susceptible and non-susceptible isolates, respectively (15). Despite the epidemiologic cutoff value being only 8 μg/mL, chlorhexidine gluconate is commonly used at dramatically higher concentrations, such as 40,000 μg/mL, to treat canine staphylococcal pyoderma (16). This extreme difference in laboratory cutoff value and clinically applied concentration results in difficult to interpret *in-vitro* test results and is plausibly one of the reasons chlorhexidine topical therapy is highly effective for treatment of canine skin infections, including antibiotic resistant infections (16).

In this study, we aimed to generate antibiogram profiles using pathogenic staphylococcal isolates from canine pyoderma cases in multiple veterinary facilities in northern Mississippi areas, which to the author’s knowledge, is the first study of this type in the North America.

## 2 Materials and methods

### 2.1 Sample collection

A total of 12 facilities located in northern Mississippi participated in the study, including 10 general practice veterinary clinics, 1 veterinary teaching hospital, and a shelter medicine facility. Facilities were enrolled by contact via phone call. Sampling kits were distributed in January 2021 to each facility, which included a client informed consent form (IACUC-20-466, Mississippi State University), patient medical history form with questions to confirm meeting inclusion criteria and sampling site, large sized exam gloves, a sterile 20 gauge needle (for rupturing a pustule during sampling), bacterial transport swab/media (Starswab II, Starplex Scientific, Ontario, Canada), and a box with pre-paid United Parcel Service (UPS) shipping label. Facilities within the city limits of Starkville, MS did not use shipping boxes; the research team picked up samples directly from these facilities. Fifteen kits were distributed to each facility, and facilities within the city limits of Starkville were supplied with additional kits, as needed. Study inclusion criteria were included as check boxes for the veterinarian to check on the sample submission form, of which all criteria were required for participation in the study. Inclusion criteria included

- Canine
- Clinical signs/lesions severe enough to warrant systemic and/or topical antibiotic therapy
- Clinical signs/lesions consistent with a diagnosis of superficial bacterial pyoderma, folliculitis, or most dermatitis (hot spot)
- No known history of recurrent, refractory, or antibiotic-resistant skin infections
- No history of systemic or topical antibiotic therapy in the previous 6 months

Veterinarians were responsible for sample collection and instructed to wear exam gloves during the process to reduce contamination. Bacterial culture samples were taken following recommendations by the Antimicrobial Guidelines Working Group of the ISCAID (previously un-ruptured pustule, beneath a crust, or epidermal collarette) (12). Practitioners were instructed to collect from a previously un-ruptured pustule, when possible. Samples were submitted via pre-paid UPS ground shipping (1-day delivery), which resulted in most samples arriving at the diagnostic laboratory within 48 h of collection. Staphylococcal swab samples are stable at room temperature in transport media for up to 7 days, so all swabs were received within an acceptable time-frame (17).

### 2.2 Bacterial identification and antibiotic susceptibility test

All cultures were submitted to the microbiology laboratory at Mississippi State University College of Veterinary Medicine. This laboratory is fully accredited by the American Association of Veterinary Laboratory Diagnosticians (AAVLD). Samples were processed utilizing standard laboratory culture methods. Each colony isolated was phenotypically identified following the manufacturer’s protocol using the Sensititre ARIS™ 2X (Thermo Fisher Scientific, Oakwood Village, Ohio, United States) system with gram positive ID plates. Bacterial species were classified via conventional taxonomy for the purposes of this study. Isolates were then tested using the companion animal gram positive MIC plate and interpreted according to the manufacturer’s and up-to-date Clinical and Laboratory Standards Institute’s (CLSI) animal guidelines available at the time of testing (18). Antibiotics included in the analysis include amikacin, amox/clav acid, ampicillin, cefazolin, cefovecin, cefpodoxime, cephalothin (surrogate for cephalexin), chloramphenicol, clindamycin, enrofloxacin, erythromycin, gentamicin, marbofloxacin, oxacillin, penicillin, rifampin, and trimethoprim/sulfa. Doxycycline was tested, but it was excluded from the analysis because the MIC test range made interpretation impossible. Oxacillin is not a clinically utilized antibiotic, but it is included in tests as a screening for methicillin resistance.

### 2.3 MIC interpretation

The MIC test range was appropriate for breakpoints available at the time of the study for antibiotics included in the analysis, but new breakpoints have been recently developed and published by CLSI for chloramphenicol, enrofloxacin, and marbofloxacin (19). Susceptibility interpretations using the newly revised breakpoints for these antibiotics was provided in a separate table. In addition to new breakpoints, the intermediate interpretive category is being renamed “susceptible dose dependent” for fluoroquinolones. The MIC range tested for each of the antibiotics with new breakpoints is not appropriate (breakpoint is below the MIC range), so interpretive category determination is impossible for isolates with an MIC value less than or equal to the lowest MIC tested. So, isolates are reported as either not interpreted (NI) or resistant, since it is impossible to determine if an isolate is susceptible, given the incorrect MIC test range.

### 2.4 Chlorhexidine susceptibility tests

Chlorhexidine susceptibility was determined by measuring the MIC and comparing it to the epidemiologic cutoff value of 8 μg/mL for the first 49 isolates collected during the study (15). Testing was conducted following CLSI microdilution protocols (20). In summary, isolates were suspended in 0.9% saline at a 0.5 McFarland turbidity standard. The suspension was further diluted 1:20 into cation adjusted Mueller Hinton broth (Becton, Dickinson and Company, New Jersey, United States). A 96-well round bottom plate was set up with a serial dilution of chlorhexidine (Duravet, Blue Springs, MO, United States) from 0.25 μg/mL to 128 μg/mL in 190 μL per well, with positive (medium and bacteria) and negative growth control (medium only). Each well was inoculated with 10 μL of bacterial suspension and incubated at 37°C for 24 h. The MIC was determined by recording the lowest concentration without visible bacterial growth.

### 2.5 D-tests

Any isolate that was susceptible to clindamycin but had a reduced susceptibility to erythromycin was tested for inducible resistance with a D-test (21). In summary, a standard Kirby-Bauer plate was prepared according to CLSI recommendations, and a clindamycin disk was placed 18 mm from an erythromycin disk (Becton, Dickinson and Company, New Jersey, United States). Inducible resistance was considered present if any isolate that had a “D” shaped zone of inhibition around the clindamycin disk, and any isolate that had circular zone of inhibition was considered fully susceptible.

#### 2.5.1 Antibiogram profiling and statistical analyses

An antibiogram was compiled for pathogenic staphylococcal isolates following the CLSI M39 document, as applicable (22). In summary, the antibiogram included clinical samples (not surveillance), only routinely tested antibiotics (and chlorhexidine), calculations based on % susceptible (intermediate results not considered susceptible), and a minimum of 30 isolates. Antibiotics included in the analysis include amikacin, amox/clav acid, ampicillin, cefazolin, cefovecin, cefpodoxime, cephalothin (surrogate for cephalexin), chloramphenicol, clindamycin, enrofloxacin, erythromycin, gentamicin, marbofloxacin, oxacillin, penicillin, rifampin, and trimethoprim/sulfa. Descriptive statistics in the antibiogram for each antibiotic included the breakpoints used, percent of isolates susceptible, MIC that inhibited 50 and 90% of isolates (MIC50, MIC90), range of MIC values detected, and the range of dilutions tested are reported. Coagulase-negative *Staphylococcus* spp. and other bacteria incidentally detected were not included in the antibiogram, since these isolates are not primary skin pathogens and are likely contaminants or non-pathogenic. Additional descriptive statistics include descriptions of lesion type and sampling site.

## 3 Results

Sampling kits were received from 75 cases and processed by the diagnostic laboratory. Nine of the 12 facilities that agreed to participate submitted sampling kits, ranging from 2–42 samples per facility. The facility that submitted 42 samples was the MSU-CVM Shelter Medicine Service, and the animals were from a variety of physical locations across northern Mississippi. A pathogenic staphylococcal isolate was detected and characterized from 67 of the cases (62 *S. pseudintermdius* and 5 *S. schleiferi*). Bacterial species with unknown or unlikely association with canine skin infections were detected in the 8 cases that did not have a pathogenic staphylococcal species. Four cases included suspected environmental contaminants (e.g., *Pseudomonas stutzeri, Bacillus* spp), three cases included coagulase-negative staphylococcal species unlikely to be pathogenic (e.g., *Staphylococcus xylosus*), and one case included *Streptococcus canis*, which is of unknown pathogenicity in canine skin infections.

Pustules, epidermal collarettes, or erosions were reported in 74 of 75 cases. One case reported alopecia as the only lesion type, but the veterinarian indicated clinical signs/lesions consistent with a diagnosis of superficial bacterial folliculitis or moist dermatitis, nevertheless. However, this was one of the 8 cases without a pathogenic staphylococcal species detected. The sampling sites included contents of an unruptured pustule (n = 36), under crust/epidermal collarette (n = 13), moist skin surface (n = 8), dry skin surface (n = 12), multiple sample sites (n = 5), and one case that did not report the sample site.

All 67 pathogenic staphylococcal isolates tested were susceptible (95% CI; 0.95–1) to clindamycin, cephalexin, and amoxicillin/clavulanic acid, which are the recommended first-line systemic antibiotics for canine skin infections (Table 1) (12). Two isolates had an intermediate MIC to erythromycin, but they were fully susceptible to clindamycin and erythromycin on a D-test. The zone of inhibition around the erythromycin disk was 27 mm for both isolates, which suggests the most appropriate interpretive category is susceptible, rather than intermediate (23). Other antibiotics showed high susceptibility rates among isolates. All isolates (100%) were susceptible to amikacin, cefazolin, cefovecin, cefpodoxime, gentamicin, oxacillin, and rifampin. Trimethoprim/sulfa demonstrated a susceptibility rate of 97%, while enrofloxacin and marbofloxacin had susceptibility rates of 98.5%, based on the breakpoints available at the time of the study. However, if the newly updated breakpoints are used, at least 9 and 73% of isolates have reduced susceptibility (either susceptible dose dependent or resistant) to enrofloxacin and marbofloxacin, respectively. Similarly, 100% of isolates were susceptible to chloramphenicol with breakpoints available at the time the study was conducted, but at least 51% of isolates have reduced susceptibility if the newly revised breakpoint is used (S ≤ 2 μg/mL) (Table 2). Considering the MIC90 for enrofloxacin, marbofloxacin, and chloramphenicol all greatly exceed the antibiotic’s susceptible breakpoint, these antibiotics are unlikely to be effective for treating staphylococcal skin infections empirically. All isolates tested (n = 49) were susceptible to chlorhexidine (Table 3).

**Table 1:** Antibiogram profiles of pathogenic staphylococcal spp. isolated from canine pyoderma.

Antimicrobial	N	R	I	S	S BP	%S	MIC50	MIC90	MICRange	Test range
Amikacin	67	0	0	67	≤4	100	≤ 4	≤ 4	≤4–≤4	4–32
Amox/Clav Acid	67	0	0	67	≤0.25	100	≤0.12	≤0.12	≤0.12–≤0.12	0.12–1
Ampicillin	67	8	0	59	≤0.25	88.1	≤0.12	0.5	≤0.12–>1	0.12–1
Cefazolin	67	0	0	67	≤2	100	≤1	≤1	≤1–≤1	1–8
Cefovecin	67	0	0	67	≤0.5	100	≤0.25	≤0.25	≤0.25–0.5	0.25–4
Cefpodoxime	67	0	0	67	≤2	100	≤2	≤2	≤2–≤2	2–16
Cephalothin	67	0	0	67	≤2	100	≤2	≤2	≤2–≤2	2–8
Chloramphenicol	67	0	0	67	≤8	100	8	8	≤4–8	4–16
Clindamycin	67	0	0	67	≤0.5	100	≤ 0.5	≤ 0.5	≤0.5–≤0.5	0.5–4
Enrofloxacin	67	1	0	66	≤0.5	98.5	≤ 0.25	≤ 0.5	≤0.25–>2	0.25–2
Erythromycin	67	0	2	65	≤0.5	97	≤ 0.5	≤ 0.5	≤0.5–1	0.5–4
Gentamicin	67	0	0	67	≤4	100	≤ 1	≤ 1	≤1–≤1	1–8
Marbofloxacin	67	1	0	66	≤1	98.5	0.5	0.5	≤0.25–>2	0.25–2
Oxacillin	67	0	0	67	≤0.25	100	≤0.25	≤0.25	≤0.25–≤0.25	0.25–4
Penicillin	67	13	0	54	≤0.12	80.1	≤0.06	2	≤0.06–>8	0.06–8
Rifampin	67	0	0	67	≤1	100	≤ 1	≤ 1	≤1–≤1	1–2
Trim/Sulfa	67	2	0	65	≤2	97	≤ 0.5	≤ 0.5	≤0.5–>2	0.5–2

N, Number of isolates; R, Resistant; I, Intermediate; S, Susceptible; S BP, Susceptible Breakpoint; %S, percent of isolates susceptible; MIC50, antibiotic concentration that inhibited ≥ 50% of isolates; MIC90, antibiotic concentration that inhibited ≥ 90% of isolates; MIC Range, range of MICs detected for the isolates included in the study; Test Range, range of antibiotic concentrations tested; all antibiotic concentration units, μg/mL.

**Table 2:** Antibiotics with recently updated breakpoint (BP).

Antimicrobial	Previous susceptible BP(18)	Updated susceptible BP(19)	%NI	%RS	MIC50	MIC90
Chloramphenicol	≤8	≤2	49	51	8	8
Enrofloxacin	≤0.5	≤0.06	81	9	≤ 0.25	≤ 0.5
Marbofloxacin	≤1	≤0.12	27	73	0.5	0.5

%NI, percent non-interpreted; %RS, percent of isolates with reduced susceptibility; MIC50, antibiotic concentration that inhibited ≥ 50% of isolates, MIC90, antibiotic concentration that inhibited ≥ 90% of isolates; all antibiotic concentration units, μg/mL.

**Table 3:** Antibiogram profile of chlorhexidine for a subset of 49 isolates.

Antimicrobial	N	R	S	S EC	%S	MIC50	MIC90	MIC range	Test range
Chlorhexidine	49	0	49	≤8	100	≤ 2	≤ 2	≤1–≤4	0.25–128

N, Number of isolates; R, Resistant; S, Susceptible; S EC, Susceptible Epidemiologic Cutoff Value; %S, percent of isolates susceptible; MIC50, antibiotic concentration that inhibited ≥ 50% of isolates; MIC90, antibiotic concentration that inhibited ≥ 90% of isolates; MIC Range, range of MICs detected for the isolates included in the study; Test Range, range of antibiotic concentrations tested; all antibiotic concentration units, μg/mL.

## 4 Discussion

The findings of this study strongly indicate that in the absence of known risk factors for resistance (e.g., recent antibiotic use or previous resistant infections), dogs with superficial bacterial folliculitis are unlikely to have an antibiotic-resistant infection. In these patients, topical therapy with chlorhexidine should be considered first line therapy, and when systemic therapy is indicated, clindamycin should be considered first line therapy. Practitioners should include prior antibiotic use and resistant infection history as standard history questions when evaluating canine patients and use this information when estimating the risk of antibiotic resistance.

Considering the reported 100% susceptibility to commonly recommended first-line systemic therapies and chlorhexidine in this study, the veterinary practitioner may have several different antimicrobials to select from and still expect a positive treatment outcome when patients are low risk for antibiotic resistance. A core principle of clinical antibiotic stewardship is avoiding unnecessary antimicrobial use, particularly systemic antibiotics. The findings in this study advocate for topical antimicrobial treatments in all cases of superficial bacterial folliculitis amenable to topical therapy, as resistance to the potent concentrations of antimicrobials used in these therapies is rare (12).

In clinical cases that systemic antibiotics are clinically indicated for pyoderma, e.g., infections that are progressing rapidly and causing significant pain, first-line antibiotics such as clindamycin, cephalexin, or amoxcillin/clavulanic acid can be considered (12, 24). Cephalosporins and potentiated penicillins, such as cephalexin and amoxicillin/clavulanic are known to select for antibiotic resistance among bystander opportunistic Enterobacterales, such as *E. coli* and *Klebsiella pneumoniae*, while clindamycin and other lincosamides have little-to-no effect on these species of bacteria (4, 14, 25, 26). Considering the high rates of ESBL/ampC Enterobacterale carriage and recent emergence of carbapenemase producing Enterobacterales in veterinary medicine, it is extremely prudent to avoid unnecessary selection pressure for these types of resistance (27–29). Additionally, clindamycin is less likely to select for methicillin resistant *Staphylococcus*, compared to cephalosporin alternatives (4). Owing to zero detected resistance in this population of dogs (including no detected inducible resistance) and less selection bacterial resistance, clindamycin should be strongly considered as first-line therapy in patients at low risk for resistance when systemic antibiotics are indicated. Considering the pharmacokinetic properties of clindamycin in dogs, the authors’ preferred dose for canine pyoderma is 11–15 mg/kg by mouth q12 hours (30).

The recent breakpoint changes for chloramphenicol, enrofloxacin, and marbofloxacin have major implications for therapy of superficial bacterial folliculitis in dogs (23, 31). When interpreting susceptibility based on the newly updated breakpoint for these antibiotics, our data suggest that fluroquinolones and chloramphenicol are unlikely to be effective against pathogenic staphylococcal isolates in superficial bacterial skin infections, even when the isolate is susceptible to most other antibiotics. Therefore, these antibiotics should not be used empirically in patients at low risk for resistance.

The antibiogram profiles generated in this study could provide practitioners with the most effective antibiotic choices, including topical therapy, for canine staphylococcal pyoderma in patients at low risk for resistance. Furthermore, it will fit the fundamental aspect of clinical antibiotic stewardship by curbing the use of antibiotics, especially unnecessary systemic medications. Antibiograms should be routinely compiled for other subgroups of patients (e.g., referral patients) and geographic locations. It is also important to continuously compile antibiograms for each subgroup, as resistance patterns may change over time.

## Data Availability

The original contributions presented in the study are included in the article/supplementary material, further inquiries can be directed to the corresponding author.
