# Correction: Staphylococcal skin infection isolates from dogs without recent antibiotic exposure are 100% susceptible to clindamycin

**DOI:** 10.3389/fvets.2025.1665861

**Published:** 2025-08-08

**Authors:** W. Cooper Brookshire, Larry D. Ballard, Vernon C. Langston, Joo Youn Park, Keun-Seok Seo

**Affiliations:** ^1^Department of Clinical Sciences, College of Veterinary Medicine, Mississippi State University, Mississippi State, MS, United States; ^2^Department of Pathobiology and Population Medicine, College of Veterinary Medicine, Mississippi State University, Mississippi State, MS, United States; ^3^Department of Comparative Biomedical Sciences, College of Veterinary Medicine, Mississippi State University, Mississippi State, MS, United States

**Keywords:** veterinary, antibiogram, *Staphylococcus pseudintermedius*, *S. schleiferi* antibiotic resistance, bacterial folliculitis, pyoderma, canine

In the published article, there was a mistake in the Funding statement. The funding statement for National Institute of General Medical Sciences of the National Institutes of Health under Award Number U54GM115428 was excluded in error. The correct funding statement is “The author(s) declare that financial support was received for the research and/or publication of this article. This study was funded by the Mississippi State University College of Veterinary Medicine Office of Research and Graduate Studies. This work was also supported by the National Institute of General Medical Sciences of the National Institutes of Health under Award Number U54GM115428.”

The original article has been updated.

